# Genome Sequence of *Xanthomonas campestris* pv. campestris Strain Xca5

**DOI:** 10.1128/genomeA.00032-12

**Published:** 2013-02-07

**Authors:** Stéphanie Bolot, Endrick Guy, Sébastien Carrere, Valérie Barbe, Matthieu Arlat, Laurent D. Noël

**Affiliations:** a National Institute for Agricultural Research (INRA), Laboratoire des Interactions Plantes-Microorganismes (LIPM), UMR 441, Castanet-Tolosan, France; b Centre National de la Recherche Scientifique (CNRS), LIPM, UMR 2594, Castanet-Tolosan, France; c Commissariat à l’Énergie Atomique et aux Energies Alternatives (CEA), IG, Genoscope, Évry, France; d Université de Toulouse, Université Paul Sabatier, Toulouse, France

## Abstract

An annotated high-quality draft genome sequence for *Xanthomonas campestris* pv. campestris race 1 strain Xca5 (originally described as *X. campestris* pv. armoraciae), the causal agent of black rot on *Brassicaceae* plants, has been determined. This genome sequence is a valuable resource for comparative genomics within the campestris pathovar.

## GENOME ANNOUNCEMENT

*Xanthomonas campestris* pv. campestris is the causal agent of black rot on a wide range of *Brassicaceae* plants, including vegetable crops, such as cabbages, ornamental crucifers, and weeds, as well as the model plant *Arabidopsis thaliana*. The bacteria are seed transmitted and enter the plant vascular system by the hydathodes, causing V-shaped lesions, vein blackening, and leaf tissue necrosis. *X. campestris* pv. campestris strains have been sorted into 9 physiological races based on their interactions with diverse *Brassicaceae* plants. *X. campestris* pv. campestris genome sequences for races 3 and 9 are available (1, 2). Yet, no strains from *X. campestris* pv. campestris race 1 have had their genome sequences determined, despite belonging to probably the most represented *X. campestris* pv. campestris race worldwide (3).

Xca5 is an American race 1 *X. campestris* pv. campestris strain originally classified as pathovar armoraciae by M. Daniels (Sainsbury Laboratory, Norwich, United Kingdom). Yet, pathogenicity tests (3), as well as multilocus sequence analyses (data not shown), clearly identified this strain as a bona fide *X. campestris* pv. campestris strain. Xca5 genome shotgun sequencing was performed on a GAIIx Illumina platform. A total of 28,569,308 76-bp paired-end reads corresponding to 4,342,534,816 bp and 868-fold coverage were obtained. Genome assembly was performed using a combination of Short Oligonucleotide Analysis Package (SOAP) *de novo* (4) and Velvet (5) assemblers and yielded 130 contigs that were >500 bp and had an *N*_50_ of 121,265 bp. The average contig size was 38,385 bp, and the largest was 447,475 bp long, for a total genome size of 4,990,056 bp. One hundred twenty of those contigs were further organized into 4 pseudomolecules. The largest one corresponds to the chromosome (4,905,337 bp; 65.2% G+C content) based on *X. campestris* pv. campestris 8004 chromosomal organization. The remaining pseudomolecules match known *Xanthomonas* plasmid sequences. Due to the highly repetitive nature of the structures of known transcriptional activator-like (TAL) protein-encoding loci *hax2* (plasmid borne), *hax3*, and *hax4*, their sequences (accession no. AY993937, AY993938, and AY993939, respectively) could not be assembled automatically (6) and were manually added to the final submission. In total, the genome is composed of 17 pseudomolecules/contigs totaling 5,001,025 bp. Annotation transfer was performed using the Rapid Annotation Transfer Tool (RATT) (7) with *X. campestris* pv. campestris B100, 8004, and ATCC 33913 as references. *De novo* annotation was performed on remaining areas using FrameD (8) and was inspected manually. We identified 4,592 coding sequences (CDSs), 51 tRNA genes, and 2 rRNA genes.

Phylogenetic analyses based on the core genome sequence shared with the 3 available *X. campestris* pv. campestris reference genome sequences and the genome sequence of *X. campestris* pv. raphani strain 756C were performed using Unus (9). These analyses showed that, indeed, Xca5 is most closely related to *X. campestris* pv. campestris strains 8004 and ATCC 33913. Using OrthoMCL (percent match cutoff, 80; blast parameter, F = false) (10), Xca5 was found to share 3,711 CDSs with the 3 *X. campestris* pv. campestris reference strains. Despite the availability of the 3 *X. campestris* pv. campestris reference genome sequences, this genome sequence represents the first *X. campestris* pv. campestris strain with TAL protein genes and plasmids, suggesting that much more genomic diversity might be expected at the intraspecific level than is anticipated from the available *X. campestris* pv. campestris genome sequences (1, 2, 11).

### Nucleotide sequence accession numbers.

The Whole Genome Shotgun project has been deposited at EMBL under the accession no. CAOR01000001 to CAOR01000130.
